# Supplementary material for: Assessing the burden of severe nausea and vomiting of pregnancy or hyperemesis gravidarum and the associated use and experiences of medication treatments: An Australian consumer survey
Source: PLoS One. 2025 Sep 3;20(9):e0329687. doi: 10.1371/journal.pone.0329687 (PMC12407409; doi:10.1371/journal.pone.0329687)
Supplement: S1 Appendix — (PDF) [file pone.0329687.s001.pdf]

Supplementary Files

Assessing the burden of severe nausea and vomiting of pregnancy or hyperemesis gravidarum and the associated use and experiences of medication treatments: an Australian consumer survey

Loyola Wills, Han-Fang Hsiao, Alicia Thomas, Caitlin Kay-Smith, Amanda Henry, Luke E Grzeskowiak

S1 Appendix – Hyperemesis Survey

Treating Severe Nausea and Vomiting in Pregnancy

Please complete the survey below.

Thank you!

Participant Information Sheet
HUMAN RESEARCH ETHICS COMMITTEE APPROVAL NUMBER: H-2020-090
PRINCIPAL INVESTIGATOR: Dr Luke Grzeskowiak

What is the project about?
We are conducting a survey to better understand what women know and have experienced regarding the use of medicines or other substances (such as herbs or vitamins) to treat severe nausea and vomiting during pregnancy or Hyperemesis Gravidarum.
Who can and cannot complete this survey?
This survey is for women in Australia who have experienced severe nausea and vomiting in pregnancy (NVP) or Hyperemesis Gravidarum. Please note that there is no fixed definition for severe nausea and vomiting in pregnancy of Hyperemesis Gravidarum. For this study, we define the condition as symptoms of nausea, vomiting and/or dry-retching commencing in pregnancy without another cause that prevents adequate intake of food and fluids and negatively impacts on physical or mental wellbeing.
The following people are not eligible to take part:
- **If you are under 18 years old**
- **If you did not receive treatment in Australia**

What am I being asked to do and how long will it take?
We ask that you complete the following survey that consists of a series of questions about yourself, demographics, your experience of nausea and vomiting during pregnancy or Hyperemesis Gravidarum, and your awareness and use (if relevant) of medications or other substances to treat symptoms of severe nausea and vomiting in pregnancy or Hyperemesis Gravidarum.
How much time will my involvement in the project take?
We estimate the survey will take 15-30 minutes to complete.
Are there any risks associated in participating in this study?
We recognise that asking questions about your pregnancy experiences could be distressing to some women. The following support services are available if you need:
Mental Health Support
Beyond Blue - 1300 224 636
Lifeline - 13 11 14
Bereavement Support
SANDS - Miscarriage, stillbirth, and newborn death support - 1300 072 637

What are the potential benefits of the research project?
There are no immediate benefits to you for participating in this study. The information you provide could help us understand how to better support women when they are making decisions about whether to take a medication or other substance to manage severe nausea and vomiting in pregnancy.

Can I withdraw from the project?

Completing the survey is voluntary. Because your survey response is completely anonymous it will not be possible to withdraw your data once responses have been submitted.

Who is undertaking the project?

This project is being conducted by Dr Luke Grzeskowiak from the University of Adelaide, Ms Alicia Thomas and Ms Han-Fang Hsiao from SA Pharmacy.

What will happen to the data I provide?

Confidentiality and privacy: Responses are completely anonymous.

Storage: All Electronic records will be kept in password protected files on the University servers. Only those directly involved in the study will have access to the data. Data will be kept for 5 years.

Publishing: Information from this project will be used to generate a report, which will be presented at a conference and submitted for publication in a peer-reviewed journal. A summary report of research findings will also be shared with members of Hyperemesis Gravidarum Australia and Hyperemesis Gravidarum Support Australia

Sharing: Individual anonymous responses to the survey will be made available to other researchers on request for research projects that are in the same general area of research. Information collected during the survey may also be used by the same researchers in future research projects that are an extension of, or closely related to, the original project.

Who do I contact if I have questions about the project?

If you have any questions about the project you can contact the following member of the research team:

Dr Luke Grzeskowiak - Email: luke.grzeskowiak@adelaide.edu.au OR Phone: (08) 8313 1687

What if I have a complaint or any concerns?

The study has been approved by the Human Research Ethics Committee at the University of Adelaide (approval number H-2020-090). This research project will be conducted according to the NHMRC National Statement on Ethical Conduct in Human Research 2007 (Updated 2018). If you have questions or problems associated with the practical aspects of your participation in the project, or wish to raise a concern or complaint about the project, then you should consult the Principal Investigator. If you wish to speak with an independent person regarding concerns or a complaint, the University's policy on research involving human participants, or your rights as a participant, please contact the Human Research Ethics Committee's Secretariat on:

Phone: +61 8 8313 6028

Email: hrec@adelaide.edu.au

Post: Level 4, Rundle Mall Plaza, 50 Rundle Mall, ADELAIDE SA 5000

Any complaint or concern will be treated in confidence and fully investigated. You will be informed of the outcome.

Yours sincerely,

Dr Luke Grzeskowiak

Ms Alicia Thomas

Ms Han-Fang Hsiao

Ver. 2.0

Date: 03/06/2020

If I want to participate, what do I do?
If you would like to participate in the study, continue to the next page. Submission of completed responses to the survey will be taken as an indication of consent to participate.

Section One

What is your current age?

____

Do you currently live in Australia?

◯ Yes
◯ No

In which State/Territory do you live?

____

Which racial or ethnic group do you belong to?

◯ Caucasian (European, Middle Eastern or Arabic)
◯ Indigenous
◯ African (Black African ancestry)
◯ North East Asian (Chinese, Japanese, Korean, Mongolian, or Tibetan etc. ancestry)
◯ South East Asian (Burmese, Thai, Lao, Vietnamese, Filipino, Indonesia, Malay etc.)
◯ Southern and Central Asian (Indian, Pakistan, Sri Lankan, Afghanistan etc.)
◯ Other
◯ Not sure/ prefer not to say

If you selected Indigenous, are you of Aboriginal or Torres Strait Islander origin?

◯ Aboriginal
◯ Torres Strait Islander
◯ Aboriginal and Torres Strait Islander
◯ Not sure/ prefer not to say

If you selected other, please specify which racial or ethnic group you belong to.

____

What is your marital status?

◯ Married/living together with partner
◯ Single
◯ In a relationship
◯ Separated/divorced
◯ Other

Did you complete secondary school? (year 12 or equivalent)?

◯ Yes
◯ No
◯ Not sure

Did you complete any further study or formal qualifications?

◯ Yes - Certificate/Apprenticeship
◯ Yes - Diploma
◯ Yes - Degree (undergraduate or honours)
◯ Yes - Higher Degree (masters or doctorate)
◯ Yes - Other
◯ No
◯ Not Sure

If you selected other, please specify the further study or formal qualifications you completed.

____

Do you have any chronic diseases (tick all that apply)?

- ☐ Allergies
- ☐ Asthma
- ☐ Diabetes (type 1 or 2)
- ☐ Epilepsy
- ☐ Cardiovascular disease
- ☐ Musculoskeletal disorders
- ☐ Hypo- or hyperthyroidism
- ☐ Depression/anxiety
- ☐ Migraine
- ☐ Other
- ☐ None

If you selected other, please specify what chronic disease you have. ______________________

How many times have you been pregnant (including miscarriages and terminations)? ______________________

How many times have you given birth? ______________________

Section Two

Which of the following have you heard can be used to manage severe nausea and vomiting in pregnancy? (tick all that apply)	☐ Ginger<br>☐ Pyridoxine (vitamin B6 [e.g. Pyridox®])<br>☐ Doxylamine (e.g. Restavit®)<br>☐ Cyclizine (e.g. Nausicalm®)<br>☐ Promethazine (e.g. Phenergan®)<br>☐ Prochlorperazine (e.g. Stemetil®, Nausetil®)<br>☐ Ondansetron (e.g. Zofran®)<br>☐ Metoclopramide (e.g. Maxolon®, Pramin®)<br>☐ Domperidone (e.g. Motilium®)<br>☐ Mirtazapine (e.g. Avanza®)<br>☐ Steroids (e.g. prednisolone [Panafcortelone®, Solone])<br>☐ Cannabis/marijuana<br>☐ Antacids (e.g. Mylanta®, Gaviscon®)<br>☐ H2 antagonist (e.g. ranitidine [Zantac™])<br>☐ Proton pump inhibitor (e.g. omeprazole [Losec®, Acimax®)<br>☐ Acupressure (e.g. SeaBand®)<br>☐ Acupuncture<br>☐ Homeopathy<br>☐ Aromatherapy<br>☐ Multivitamins (combination of vitamins)<br>☐ Herbal remedies<br>☐ Intravenous fluids/hydration<br>☐ Vitamin B1 (thiamine)<br>☐ Nasogastric (tube) feeds<br>☐ Other<br>☐ None
If you selected other, please specify what you have heard can be used to manage severe nausea and vomiting in pregnancy.	__________________________

How safe do you think each of the following are for mothers when used to manage severe nausea and vomiting in pregnancy? (1 being not very safe, 5 being very safe)
1 = Not Very Safe
2 = Somewhat Unsafe
3 = Not Sure
4 = Somewhat Safe
5 = Very Safe

	1	2	3	4	5
Ginger	○	○	○	○	○
Pyridoxine (vitamin B6 [e.g. Pyridox®)	○	○	○	○	○
Doxylamine (e.g. Restavit®)	○	○	○	○	○
Cyclizine (e.g. Nausicalm)	○	○	○	○	○
Promethazine (e.g. Phenergan®)	○	○	○	○	○
Prochlorperazine (e.g. Stemetil, Nausetil)	○	○	○	○	○
Ondansetron (e.g. Zofran®)	○	○	○	○	○
Metoclopramide (e.g. Maxolon®, Pramin®)	○	○	○	○	○
Domperidone (e.g. Motilium®)	○	○	○	○	○
Mirtazapine (e.g. Avanza®)	○	○	○	○	○
Steroids (e.g. prednisolone [Panafcortelone®, Solone])	○	○	○	○	○
Cannabis/marijuana	○	○	○	○	○
Antacids (e.g. Mylanta®, Gaviscon®)	○	○	○	○	○
H2 antagonist (e.g. ranitidine [Zantac™])	○	○	○	○	○
Proton pump inhibitor (e.g. omeprazole [Losec®, Acimax®])	○	○	○	○	○
Acupressure (e.g. SeaBand®)	○	○	○	○	○
Acupuncture	○	○	○	○	○
Homeopathy	○	○	○	○	○
Aromatherapy	○	○	○	○	○
Multivitamins (combinations of vitamins)	○	○	○	○	○
Herbal Therapies	○	○	○	○	○
Intravenous fluids/hydration	○	○	○	○	○
Vitamin B1 (thiamine)	○	○	○	○	○
Nasogastric (tube) feeds	○	○	○	○	○

How safe do you think each of the following are for unborn babies when used to manage severe nausea and vomiting in pregnancy? (1 being not very safe, 5 being very safe)
1 = Not Very Safe
2 = Somewhat Unsafe
3 = Not Sure
4 = Somewhat Safe
5 = Very Safe

	1	2	3	4	5
Ginger	○	○	○	○	○
Pyridoxine (vitamin B6)	○	○	○	○	○
Doxylamine (e.g. Restavit®)	○	○	○	○	○
Cyclizine (e.g. Nausicalm)	○	○	○	○	○
Promethazine (e.g. Phenergan)	○	○	○	○	○
Prochlorperazine (e.g. Stemetil, Nausetil)	○	○	○	○	○
Ondansetron (e.g. Zofran)	○	○	○	○	○
Metoclopramide (e.g. Maxolon, Pramin)	○	○	○	○	○
Domperidone (e.g. Motilium)	○	○	○	○	○
Mirtazapine (e.g. Avanza®)	○	○	○	○	○
Steroids (e.g. prednisolone)	○	○	○	○	○
Cannabis/marijuana	○	○	○	○	○
Antacids (e.g. Mylanta, Gaviscon)	○	○	○	○	○
H2 antagonist (e.g. ranitidine [Zantac])	○	○	○	○	○
Proton pump inhibitor (e.g. omeprazole)	○	○	○	○	○
Acupressure (SeaBand)	○	○	○	○	○
Acupuncture	○	○	○	○	○
Homeopathy	○	○	○	○	○
Aromatherapy	○	○	○	○	○
Multivitamins (combinations of vitamins)	○	○	○	○	○
Herbal remedies	○	○	○	○	○
Intravenous fluids/hydration	○	○	○	○	○
Vitamin B1 (thiamine)	○	○	○	○	○
Nasogastric (tube) feeds	○	○	○	○	○

Which of the following sources have you used to obtain information on the use of medicines or other substances to manage severe nausea and vomiting in pregnancy? (tick all that apply)	☐ None<br>☐ General Practitioner (GP)<br>☐ Obstetrician/Gynaecologist<br>☐ Doctor in Emergency Department Or Hospital<br>☐ Specialist Doctor (e.g. obstetric medicine, endocrinology)<br>☐ Midwife<br>☐ Nurse<br>☐ Community Pharmacist<br>☐ Hospital Pharmacist<br>☐ Medicines Information Telephone Service (e.g. MotherSafe, NPS)<br>☐ Naturopath<br>☐ Family<br>☐ Friends<br>☐ Mother's group (i.e. face-to-face)<br>☐ Internet search (e.g. Google)<br>☐ Social media (e.g. Instagram, Twitter, Facebook)<br>☐ Blogs or Online Discussion Forums<br>☐ Other
If you selected social media, please specify your source.	☐ Reddit<br>☐ Instagram<br>☐ Facebook<br>☐ Twitter<br>☐ Snapchat<br>☐ Other
If you selected other, please specify which information source.	__________________________________

How useful were the following in helping you decide which medicine or substance to take to manage your severe nausea and vomiting during your pregnancy, on a scale of 1-5? (1 being not useful at all and 5 being very useful)
1 = Not Very Useful
2 = Somewhat Not Useful
3 = Not Sure
4 = Somewhat Useful
5 = Very Useful

	1	2	3	4	5
General Practitioner (GP)	○	○	○	○	○
Obstetrician/Gynaecologist	○	○	○	○	○
Emergency Department/Hospital Doctor	○	○	○	○	○
Specialist Physician (e.g. obstetric medicine, endocrinology)	○	○	○	○	○
Midwife	○	○	○	○	○
Nurse	○	○	○	○	○
Community Pharmacist	○	○	○	○	○
Naturopath	○	○	○	○	○
Family	○	○	○	○	○
Friends	○	○	○	○	○
Mother's Group (e.g. face-to-face)	○	○	○	○	○
Internet Search (e.g. Google)	○	○	○	○	○
Social Media (e.g. Instagram, Twitter, Facebook)	○	○	○	○	○
Blogs or Online Discussion Forums	○	○	○	○	○
Other Source You Mentioned Previously	○	○	○	○	○

Section Three

How many of your pregnancies resulted in severe nausea and vomiting or Hyperemesis Gravidarum?	◯ 1 pregnancy<br>◯ 2 pregnancies<br>◯ 3 or more pregnancies
Are you pregnant now?	◯ Yes<br>◯ No
If yes, in which week of pregnancy are you now (calculated from the first day of your last menstrual period)?	__________________________
Have you suffered from severe nausea and vomiting or Hyperemesis Gravidarum during your current pregnancy?	◯ Yes<br>◯ No
How long ago were you pregnant and suffering from severe nausea and vomiting or Hyperemesis Gravidarum (please answer with respect to most recent pregnancy)?	◯ < 6 months<br>◯ 6-12 months<br>◯ 1-2 years<br>◯ > 2 years
We ask you to answer the rest of the survey on the basis of your experiences from this pregnancy	
Please answer the following questions relating to your most recent pregnancy affected by severe nausea and vomiting or Hyperemesis Gravidarum	
How many weeks pregnant were you when the nausea and/or vomiting started?	__________________________
Are you still suffering with nausea and/or vomiting more than 3 days per week?	◯ Yes<br>◯ No
If no, then how many weeks pregnant were you when the nausea and/or vomiting improved?	__________________________
In which pregnancy week did your nausea and vomiting stop?	__________________________
Did you lose weight during pregnancy because of your severe nausea and vomiting?	◯ Yes<br>◯ No<br>◯ Not Sure
How much did you weigh prior to pregnancy?	__________________________<br>(Please state as kilograms (kg))
What was your lowest weight during your pregnancy?	__________________________<br>(Please state in kilograms (kg))
Did you take folic acid prior to becoming pregnant?	◯ Yes<br>◯ No
Did you smoke during your pregnancy AFTER you found out you were pregnant?	◯ Yes, I smoked regularly (daily)<br>◯ Yes, occasionally (less than daily)<br>◯ No, never<br>◯ Prefer not to answer

Did you drink alcohol during your pregnancy AFTER you found out you were pregnant?	◯ Yes, I drank alcohol regularly (daily)<br>◯ Yes, occasionally (less than daily)<br>◯ No, never<br>◯ Prefer not to answer

**Please think back on a typical 24 hours during the period when you symptoms were at their worst:**	
For how long did you feel nauseated or sick to your stomach?	◯ Not at all<br>◯ 1 hour or less<br>◯ 2-3 hours<br>◯ 4-6 hours<br>◯ More than 6 hours
Did you vomit or throw up?	◯ I did not throw up<br>◯ 1-2 times<br>◯ 3-4 times<br>◯ 5-6 times<br>◯ 7 or more times
How many times did you have retching or dry heaves without bringing anything up?	◯ None<br>◯ 1-2 times<br>◯ 3-4 times<br>◯ 5-6 times<br>◯ 7 or more times
How many hours did you sleep out of the 24 hours?	__________________________

**Because of your severe nausea and vomiting:**	
I was diagnosed with Hyperemesis Gravidarum?	◯ Yes<br>◯ No
I had great difficulty in eating and drinking and I was unable to eat and/or drink as much as usual.	◯ Yes<br>◯ No
I had to go to hospital and be put on a drip because I was dehydrated.	◯ Yes<br>◯ No
I took anti-sickness medication.	◯ Yes<br>◯ No
I suffered from severe nausea which was always present during waking hours.	◯ Yes<br>◯ No
How many additional visits to your GP or Specialist Doctor did you have to help manage your symptoms?	__________________________
How many times did you visit the Hospital Emergency Department?	__________________________
How many hospital admissions for severe nausea and vomiting did you have (including day or overnight admissions for treatment)?	__________________________
How many weeks pregnant were you when you received a diagnosis of Hyperemesis Gravidarum?	__________________________<br>(Weeks gestation)
Do you wish that you had been diagnosed earlier?	◯ Yes<br>◯ No

How has/did nausea and vomiting of pregnancy effected your everyday life with regards to the following aspects? (1 being not at all, 5 being major impact)

	1	2	3	4	5
Inhibition of the ability to take care of household chores	○	○	○	○	○
Reduced social life	○	○	○	○	○
Negative impact on relationship with partner	○	○	○	○	○
Reduced work capacity	○	○	○	○	○
The ability to care for any children from previous pregnancies	○	○	○	○	○
Having uninterrupted sleep	○	○	○	○	○
Inhibition of the ability to eat or drink	○	○	○	○	○

Do you/have you experienced any feeling of depression or anxiety as a result of your nausea and vomiting of pregnancy?
- ○ Never
- ○ Rarely
- ○ Sometimes
- ○ Often
- ○ Always

Did your feelings of depression or anxiety start during or after pregnancy?
- ○ During Pregnancy
- ○ After Pregnancy

Were your symptoms ever so severe that you requested an induction of labour or elective cesarean section to end the pregnancy earlier?
- ○ Yes
- ○ No

Did you end up being induced or delivering by elective cesarean section because of your severe nausea and vomiting or Hyperemesis Gravidarum?
- ○ Yes
- ○ No

Have you ever considered terminating your pregnancy due to your severe nausea and vomiting?
- ○ Yes
- ○ No
- ○ Prefer not to answer

Have you ever considered not having more children due to your severe nausea and vomiting?
- ○ Yes
- ○ No
- ○ Prefer not to answer

Attitudes towards medication use during pregnancy:

	Strongly Disagree	Disagree	Uncertain	Agree	Strongly Agree
I have a higher threshold for using medicines when I am pregnant than when I'm not pregnant	○	○	○	○	○
It is better for the fetus that I use medicines and get well than to have an untreated illness during pregnancy	○	○	○	○	○
Pregnant women should preferably use herbal remedies rather than conventional medicines	○	○	○	○	○
Even though I had nausea and vomiting of pregnancy (NVP) I chose to refrain from using medicines for NVP just to be safe	○	○	○	○	○
I was anxious about how the medicines affected the fetus	○	○	○	○	○
I used less medicine than needed for nausea due to being pregnant	○	○	○	○	○

Section Four

Which of the following did you use during pregnancy to manage severe nausea and vomiting or Hyperemesis Gravidarum? (tick all that apply)

- ☐ Ginger
- ☐ Pyridoxine (vitamin B6)
- ☐ Doxylamine (e.g. Restavit®)
- ☐ Cyclizine (e.g. Nausicalm®)
- ☐ Promethazine (e.g. Phenergan®)
- ☐ Prochlorperazine (e.g. Stemetil®, Nausetil®)
- ☐ Ondansetron (e.g. Zofran®)
- ☐ Metoclopramide (e.g. Maxolon®, Pramin®)
- ☐ Domperidone (e.g. Motilium®)
- ☐ Mirtazapine (e.g. Avanza®)
- ☐ Steroids (e.g. prednisolone [Panafcortelone®, Solone])
- ☐ Cannabis/marijuana
- ☐ Antacids (e.g. Mylanta®, Gaviscon®)
- ☐ H2 antagonist (e.g. ranitidine [Zantac®])
- ☐ Proton pump inhibitor (e.g. omeprazole [Losec®, Acimax®])
- ☐ Acupressure (SeaBand®)
- ☐ Acupuncture
- ☐ Homeopathy
- ☐ Aromatherapy
- ☐ Multivitamins (combination of vitamins)
- ☐ Herbal remedies
- ☐ Intravenous fluids/hydration
- ☐ Vitamin B1 (thiamine)
- ☐ Nasogastric (tube) feeds
- ☐ Other
- ☐ None

If you selected other, please specify what you used. __________________________

The following questions relate to your use of ginger during pregnancy for managing nausea and vomiting of pregnancy

Who recommended ginger to you?

☐ General Practitioner (GP)
☐ Obstetrician/Gynaecologist
☐ Doctor in Emergency Department or Hospital
☐ Specialist Doctor (e.g. obstetric medicine, endocrinology)
☐ Midwife
☐ Nurse
☐ Community Pharmacist
☐ Hospital Pharmacist
☐ Medicines Information Telephone Service (e.g. MotherSafe)
☐ Naturopath
☐ Family/friends
☐ Internet
☐ Social Media
☐ Other
(Select all that apply)

If you selected other, please specify who recommended ginger to you.

____

When did you start taking ginger during your pregnancy?

____
(Which week of your pregnancy?)

Are you still using ginger?

◯ Yes
◯ No

If yes, how long have you been taking ginger?

____
(How many weeks?)

If no, how long did you take ginger for?

____
(How many weeks?)

How did you take ginger?

☐ Tablet/Capsule
☐ Food
☐ Tea
(Select all that apply)

What was the highest number of tablets/capsules you took in a single day?

____

What was the approximate dose of each ginger tablet/capsule?

◯ 200 mg
◯ 400 mg
◯ 600 mg
◯ Other
◯ Don't Know

What was the dose of ginger?

____

Did you experience any of the following side effects while taking ginger? (tick all that apply)	☐ Headache<br>☐ Muscle/body weakness<br>☐ Numbness/pins-and-needles in hands or feet<br>☐ Stomach cramps<br>☐ Heartburn/indigestion<br>☐ Constipation<br>☐ Diarrhoea<br>☐ Impaired alertness, cognition, memory<br>☐ Sedation (e.g. sleepiness)<br>☐ Worsening nausea/vomiting<br>☐ Dry mouth<br>☐ Blurred vision<br>☐ Other<br>☐ None
If you selected other, please specify what side effects you experienced while taking ginger.	__________________________________
Did you stop taking ginger because of side effects?	◯ Yes<br>◯ No
Did you ever want to stop taking ginger because of side effects?	◯ Yes<br>◯ No
How effective do you feel ginger was in managing your severe nausea and vomiting in pregnancy? (1 being not very effective and 5 being very effective)	◯ 1<br>◯ 2<br>◯ 3<br>◯ 4<br>◯ 5
Would you use ginger again?	◯ Yes<br>◯ No
Would you recommend ginger to a friend?	◯ Yes<br>◯ No
If not, why would you not recommend ginger?	☐ Too expensive<br>☐ Side effects<br>☐ Does not work<br>☐ Other
If you selected other, please specify why you would not recommend ginger.	__________________________________

The following questions relate to your use of pyridoxine (vitamin B6 [e.g. Pyridox®]) during pregnancy for managing nausea and vomiting of pregnancy

Who recommended/prescribed pyridoxine (vitamin B6 [e.g. Pyridox®]) to you?	☐ General Practitioner (GP)<br>☐ Obstetrician/Gynaecologist<br>☐ Doctor in Emergency Department or Hospital<br>☐ Specialist Doctor (e.g. obstetric medicine, endocrinology)<br>☐ Midwife<br>☐ Nurse<br>☐ Community Pharmacist<br>☐ Hospital Pharmacist<br>☐ Medicines Information Telephone Service (e.g. MotherSafe)<br>☐ Naturopath<br>☐ Family/friends<br>☐ Internet<br>☐ Social Media<br>☐ Other<br>(Select all that apply)
If you selected other, please specify who recommended/prescribed pyridoxine (vitamin B6 [e.g. Pyridox®]) to you.	__________________________
When did you start taking pyridoxine (vitamin B6 [e.g. Pyridox®]) during your pregnancy?	__________________________<br>(Which week of your pregnancy?)
Are you still using pyridoxine (vitamin B6 [e.g. Pyridox®])?	◯ Yes<br>◯ No
If yes, how long have you been taking pyridoxine (vitamin B6 [e.g. Pyridox®])?	__________________________<br>(How many weeks?)
If no, how long did you take pyridoxine (vitamin B6 [e.g. Pyridox®]) for?	__________________________<br>(How many weeks?)
What was the highest number of pyridoxine (vitamin B6 [e.g. Pyridox®]) tablets/capsules that you took in a single day?	__________________________
What was the dose of each pyridoxine (vitamin B6 [e.g. Pyridox®]) capsule/tablet?	◯ 25 mg<br>◯ Other<br>◯ Don't Know
If you selected other, please specify what the dose was for each pyridoxine (vitamin B6 [e.g. Pyridox®]) capsule/tablet.	__________________________

Did you experience any of the following side effects while taking pyridoxine (vitamin B6 [e.g. Pyridox®])? (tick all that apply)	☐ Headache<br>☐ Muscle/body weakness<br>☐ Numbness/pins-and-needles in hands or feet<br>☐ Stomach cramps<br>☐ Heartburn/indigestion<br>☐ Constipation<br>☐ Diarrhoea<br>☐ Impaired alertness, cognition, memory<br>☐ Sedation (e.g. sleepiness)<br>☐ Worsening nausea/vomiting<br>☐ Dry mouth<br>☐ Blurred vision<br>☐ Other<br>☐ None
If you selected other, please specify what side effects you experienced while taking pyridoxine (vitamin B6 [e.g. Pyridox®]).	__________________________________
Did you stop taking pyridoxine (vitamin B6 [e.g. Pyridox®]) because of side effects?	◯ Yes<br>◯ No
Did you ever want to stop taking pyridoxine (vitamin B6 [e.g. Pyridox®]) because of side effects?	◯ Yes<br>◯ No
How effective do you feel pyridoxine (vitamin B6 [e.g. Pyridox®]) was in managing your severe nausea and vomiting in pregnancy? (1 being not very effective and 5 being very effective)	◯ 1<br>◯ 2<br>◯ 3<br>◯ 4<br>◯ 5
Would you use pyridoxine (vitamin B6 [e.g. Pyridox®]) again?	◯ Yes<br>◯ No
Would you recommend pyridoxine (vitamin B6 [e.g. Pyridox®]) to a friend?	◯ Yes<br>◯ No
If not, why would you not recommend pyridoxine (vitamin B6 [e.g. Pyridox®])?	☐ Too expensive<br>☐ Side effects<br>☐ Does not work<br>☐ Other
If you selected other, please specify why you would not recommend pyridoxine (vitamin B6 [e.g. Pyridox®]).	__________________________________

The following questions relate to your use of doxylamine (e.g. Restavit®) during pregnancy for managing nausea and vomiting of pregnancy

Who recommended/prescribed doxylamine (e.g. Restavit®) to you?

- ☐ General Practitioner (GP)
- ☐ Obstetrician/Gynaecologist
- ☐ Doctor in Emergency Department or Hospital
- ☐ Specialist Doctor (e.g. obstetric medicine, endocrinology)
- ☐ Midwife
- ☐ Nurse
- ☐ Community Pharmacist
- ☐ Hospital Pharmacist
- ☐ Medicines Information Telephone Service (e.g. MotherSafe)
- ☐ Naturopath
- ☐ Family/friends
- ☐ Internet
- ☐ Social Media
- ☐ Other

If you selected other, please specify who recommended/prescribed doxylamine (e.g. Restavit®) to you.

____

When did you start taking doxylamine (e.g. Restavit®) during your pregnancy?

____
(Which week of your pregnancy?)

Are you still using doxylamine (e.g. Restavit®)?

- ◯ Yes
- ◯ No

If yes, how long have you been taking doxylamine (e.g. Restavit®)?

____
(How many weeks?)

If no, how long did you take doxylamine (e.g. Restavit®) for?

____
(How many weeks?)

What was the highest number of doxylamine (e.g. Restavit®) tablets/capsules that you took in a single day?

____

What was the dose of each doxylamine (e.g. Restavit®) capsule/tablet?

- ◯ 25 mg
- ◯ Other
- ◯ Don't Know

If you selected other, please specify the dose of each doxylamine (e.g. Restavit®) capsule/tablet.

____

Did you experience any of the following side effects while taking doxylamine (e.g. Restavit®)? (tick all that apply)	☐ Headache<br>☐ Muscle/body weakness<br>☐ Numbness/pins-and-needles in hands or feet<br>☐ Stomach cramps<br>☐ Heartburn/indigestion<br>☐ Constipation<br>☐ Diarrhoea<br>☐ Impaired alertness, cognition, memory<br>☐ Sedation (e.g. sleepiness)<br>☐ Worsening nausea/vomiting<br>☐ Dry mouth<br>☐ Blurred vision<br>☐ Other<br>☐ None
If you selected other, please specify what side effects you experienced while taking doxylamine (e.g. Restavit®).	______________________________
Did you stop taking doxylamine (e.g. Restavit®) because of side effects?	◯ Yes<br>◯ No
Did you ever want to stop taking doxylamine (e.g. Restavit®) because of side effects?	◯ Yes<br>◯ No
How effective do you feel doxylamine (e.g. Restavit®) was in managing your severe nausea and vomiting in pregnancy? (1 being not very effective and 5 being very effective)	◯ 1<br>◯ 2<br>◯ 3<br>◯ 4<br>◯ 5
Would you use doxylamine (e.g. Restavit®) again?	◯ Yes<br>◯ No
Would you recommend doxylamine (e.g. Restavit®) to a friend?	◯ Yes<br>◯ No
If not, why would you not recommend doxylamine (e.g. Restavit®)?	☐ Too expensive<br>☐ Side effects<br>☐ Does not work<br>☐ Other
If you selected other, please specify why you would not recommend doxylamine (e.g. Restavit®).	______________________________

The following questions relate to your use of cyclizine (e.g. Nausicalm®) during pregnancy for managing nausea and vomiting of pregnancy

Who recommended/prescribed cyclizine (e.g. Nausicalm®) to you?

- ☐ General Practitioner (GP)
- ☐ Obstetrician/Gynaecologist
- ☐ Doctor in Emergency Department or Hospital
- ☐ Specialist Doctor (e.g. obstetric medicine, endocrinology)
- ☐ Midwife
- ☐ Nurse
- ☐ Community Pharmacist
- ☐ Hospital Pharmacist
- ☐ Medicines Information Telephone Service (e.g. MotherSafe)
- ☐ Naturopath
- ☐ Family/friends
- ☐ Internet
- ☐ Social Media
- ☐ Other

If you selected other, please specify who recommended/prescribed cyclizine (e.g. Nausicalm®) to you.

____

When did you start taking cyclizine (e.g. Nausicalm®) during your pregnancy?

____
(Which week of your pregnancy?)

Are you still using cyclizine (e.g. Nausicalm®)?

- ◯ Yes
- ◯ No

If yes, how long have you been taking cyclizine (e.g. Nausicalm®)?

____
(How many weeks?)

If no, how long did you take cyclizine (e.g. Nausicalm®) for?

____
(How many weeks?)

How did you use cyclizine (e.g. Nausicalm®)?

- ☐ Tablet/capsule
- ☐ Injection

(Select all that apply)

What was the highest number of cyclizine (e.g. Nausicalm®) tablets/capsules that you took in a single day?

____

What was the dose of each cyclizine (e.g. Nausicalm®) capsule/tablet?

- ◯ 50 mg
- ◯ Other
- ◯ Don't Know

If you selected other, please specify the dose of each cyclizine (e.g. Nausicalm®) capsule/tablet.

____

Did you experience any of the following side effects while taking cyclizine (e.g. Nausicalm®)? (tick all that apply)	☐ Headache<br>☐ Muscle/body weakness<br>☐ Numbness/pins-and-needles in hands or feet<br>☐ Stomach cramps<br>☐ Heartburn/indigestion<br>☐ Constipation<br>☐ Diarrhoea<br>☐ Impaired alertness, cognition, memory<br>☐ Sedation (e.g. sleepiness)<br>☐ Worsening nausea/vomiting<br>☐ Dry mouth<br>☐ Blurred vision<br>☐ Other<br>☐ None
If you selected other, please specify what side effects you experienced while taking cyclizine (e.g. Nausicalm®).	__________________________________
Did you stop taking cyclizine (e.g. Nausicalm®) because of side effects?	◯ Yes<br>◯ No
Did you ever want to stop taking cyclizine (e.g. Nausicalm®) because of side effects?	◯ Yes<br>◯ No
How effective do you feel cyclizine (e.g. Nausicalm®) was in managing your severe nausea and vomiting in pregnancy? (1 being not very effective and 5 being very effective)	◯ 1<br>◯ 2<br>◯ 3<br>◯ 4<br>◯ 5
Would you use cyclizine (e.g. Nausicalm®) again?	◯ Yes<br>◯ No
Would you recommend cyclizine to a friend?	◯ Yes<br>◯ No
If not, why would you not recommend cyclizine?	☐ Too expensive<br>☐ Side effects<br>☐ Does not work<br>☐ Other
If you selected other, please specify why you would not recommend cyclizine.	__________________________________

The following questions relate to your use of promethazine (e.g. Phenergan®) during pregnancy for managing nausea and vomiting of pregnancy

Who recommended/prescribed promethazine (e.g. Phenergan®) to you?

- ☐ General Practitioner (GP)
- ☐ Obstetrician/Gynaecologist
- ☐ Doctor in Emergency Department or Hospital
- ☐ Specialist Doctor (e.g. obstetric medicine, endocrinology)
- ☐ Midwife
- ☐ Nurse
- ☐ Community Pharmacist
- ☐ Hospital Pharmacist
- ☐ Medicines Information Telephone Service (e.g. MotherSafe)
- ☐ Naturopath
- ☐ Family/friends
- ☐ Internet
- ☐ Social Media
- ☐ Other

If you selected other, please specify who recommended/prescribed promethazine (e.g. Phenergan®) to you.

____

When did you start taking promethazine (e.g. Phenergan®) during your pregnancy?

____
(Which week of your pregnancy?)

Are you still using promethazine (e.g. Phenergan®)?

- ◯ Yes
- ◯ No

If yes, how long have you been taking promethazine (e.g. Phenergan®)?

____
(How many weeks?)

If no, how long did you take promethazine (e.g. Phenergan®) for?

____
(How many weeks?)

How did you use promethazine (e.g. Phenergan®)?

- ☐ Tablet/capsule
- ☐ Injection
- ☐ Suppository

(Select all that apply)

What was the highest number of promethazine (e.g. Phenergan®) tablets/capsules that you took in a single day?

____

What was the dose of each promethazine (e.g. Phenergan®) tablet/capsule?

- ◯ 10 mg
- ◯ 25 mg
- ◯ Other
- ◯ Don't Know

If you selected other, please specify the dose of each promethazine (e.g. Phenergan®) tablet/capsule.

____

Did you experience any of the following side effects while taking promethazine (e.g. Phenergan®)? (tick all that apply)	☐ Headache<br>☐ Muscle/body weakness<br>☐ Numbness/pins-and-needles in hands or feet<br>☐ Stomach cramps<br>☐ Heartburn/indigestion<br>☐ Constipation<br>☐ Diarrhoea<br>☐ Impaired alertness, cognition, memory<br>☐ Sedation (e.g. sleepiness)<br>☐ Worsening nausea/vomiting<br>☐ Dry mouth<br>☐ Blurred vision<br>☐ Other<br>☐ None
If you selected other, please specify what side effects you experienced while taking promethazine (e.g. Phenergan®).	__________________________
Did you stop taking promethazine (e.g. Phenergan®) because of side effects?	◯ Yes<br>◯ No
Did you ever want to stop taking promethazine (e.g. Phenergan®) because of side effects?	◯ Yes<br>◯ No
How effective do you feel promethazine (e.g. Phenergan®) was in managing your severe nausea and vomiting in pregnancy? (1 being not very effective and 5 being very effective)	◯ 1<br>◯ 2<br>◯ 3<br>◯ 4<br>◯ 5
Would you use promethazine (e.g. Phenergan®) again?	◯ Yes<br>◯ No
Would you recommend promethazine (e.g. Phenergan®) to a friend?	◯ Yes<br>◯ No
If not, why would you not recommend promethazine (e.g. Phenergan®)?	☐ Too expensive<br>☐ Side effects<br>☐ Does not work<br>☐ Other
If you selected other, please specify why you would not recommend promethazine (e.g. Phenergan®).	__________________________

The following questions relate to your use of prochlorperazine (e.g. Stemetil®, Nausetil®) during pregnancy for managing nausea and vomiting of pregnancy

Who recommended/prescribed prochlorperazine (e.g. Stemetil®, Nausetil®) to you?

- ☐ General Practitioner (GP)
- ☐ Obstetrician/Gynaecologist
- ☐ Doctor in Emergency Department or Hospital
- ☐ Specialist Doctor (e.g. obstetric medicine, endocrinology)
- ☐ Midwife
- ☐ Nurse
- ☐ Community Pharmacist
- ☐ Hospital Pharmacist
- ☐ Medicines Information Telephone Service (e.g. MotherSafe)
- ☐ Naturopath
- ☐ Family/friends
- ☐ Internet
- ☐ Social Media
- ☐ Other

If you selected other, please specify who recommended/prescribed prochlorperazine (e.g. Stemetil®, Nausetil®) to you.

____

When did you start taking prochlorperazine (e.g. Stemetil®, Nausetil®) during your pregnancy?

____
(Which week of your pregnancy?)

Are you still using prochlorperazine (e.g. Stemetil®, Nausetil®)?

- ◯ Yes
- ◯ No

If yes, how long have you been taking prochlorperazine (e.g. Stemetil®, Nausetil®)?

____
(How many weeks?)

If no, how long did you take prochlorperazine (e.g. Stemetil®, Nausetil®) for?

____
(How many weeks?)

How did you use prochlorperazine (e.g. Stemetil®, Nausetil®)?

- ☐ Tablet/capsule
- ☐ Injection
- ☐ Suppository

(Select all that apply)

What was the highest number of prochlorperazine (e.g. Stemetil®, Nausetil®) tablets/capsules that you took in a single day?

____

What was the dose of each prochlorperazine (e.g. Stemetil®, Nausetil®) capsule/tablet?

- ◯ 5 mg
- ◯ Other
- ◯ Don't Know

Did you experience any of the following side effects while taking prochlorperazine (e.g. Stemetil®, Nausetil®)? (tick all that apply)	☐ Headache<br>☐ Muscle/body weakness<br>☐ Numbness/pins-and-needles in hands or feet<br>☐ Stomach cramps<br>☐ Heartburn/indigestion<br>☐ Constipation<br>☐ Diarrhoea<br>☐ Impaired alertness, cognition, memory<br>☐ Sedation (e.g. sleepiness)<br>☐ Worsening nausea/vomiting<br>☐ Dry mouth<br>☐ Blurred vision<br>☐ Other<br>☐ None
If you selected other, please specify what side effects you experienced while taking prochlorperazine (e.g. Stemetil®, Nausetil®).	__________________________________
Did you stop taking prochlorperazine (e.g. Stemetil®, Nausetil®) because of side effects?	◯ Yes<br>◯ No
Did you ever want to stop taking prochlorperazine (e.g. Stemetil®, Nausetil®) because of side effects?	◯ Yes<br>◯ No
How effective do you feel prochlorperazine (e.g. Stemetil®, Nausetil®) was in managing your severe nausea and vomiting in pregnancy? (1 being not very effective and 5 being very effective)	◯ 1<br>◯ 2<br>◯ 3<br>◯ 4<br>◯ 5
Would you use prochlorperazine (e.g. Stemetil®, Nausetil®) again?	◯ Yes<br>◯ No
Would you recommend prochlorperazine (e.g. Stemetil®, Nausetil®) to a friend?	◯ Yes<br>◯ No
If not, why would you not recommend prochlorperazine (e.g. Stemetil®, Nausetil®)?	☐ Too expensive<br>☐ Side effects<br>☐ Does not work<br>☐ Other
If you selected other, please specify why you would not recommend prochlorperazine (e.g. Stemetil®, Nausetil®).	__________________________________

The following questions relate to your use of ondansetron (e.g. Zofran®) during pregnancy for managing nausea and vomiting of pregnancy

Who recommended/prescribed ondansetron (e.g. Zofran®) to you?

- ☐ General Practitioner (GP)
- ☐ Obstetrician/Gynaecologist
- ☐ Doctor in Emergency Department or Hospital
- ☐ Specialist Doctor (e.g. obstetric medicine, endocrinology)
- ☐ Midwife
- ☐ Nurse
- ☐ Community Pharmacist
- ☐ Hospital Pharmacist
- ☐ Medicines Information Telephone Service (e.g. MotherSafe)
- ☐ Naturopath
- ☐ Family/friends
- ☐ Internet
- ☐ Social Media
- ☐ Other

If you selected other, please specify who recommended/prescribed ondansetron (e.g. Zofran®) to you.

____

When did you start taking ondansetron (e.g. Zofran®) during your pregnancy?

____
(Which week of your pregnancy?)

Are you still using ondansetron (e.g. Zofran®)?

- ◯ Yes
- ◯ No

If yes, how long have you been taking ondansetron (e.g. Zofran®)?

____
(How many weeks?)

If no, how long did you take ondansetron (e.g. Zofran®) for?

____
(How many weeks?)

How did you use ondansetron (e.g. Zofran®)?

- ☐ Tablet/capsule/wafer
- ☐ Injection
- ☐ Liquid

(Select all that apply)

What was the highest number of ondansetron (e.g. Zofran®) tablets/capsules that you took in a single day?

____

What was the dose of each ondansetron (e.g. Zofran®) tablet/capsule?

- ◯ 4 mg
- ◯ 8 mg
- ◯ Don't Know

Did you experience any of the following side effects while taking ondansetron (e.g. Zofran®)? (tick all that apply)	☐ Headache<br>☐ Muscle/body weakness<br>☐ Numbness/pins-and-needles in hands or feet<br>☐ Stomach cramps<br>☐ Heartburn/indigestion<br>☐ Constipation<br>☐ Diarrhoea<br>☐ Impaired alertness, cognition, memory<br>☐ Sedation (e.g. sleepiness)<br>☐ Worsening nausea/vomiting<br>☐ Dry mouth<br>☐ Blurred vision<br>☐ Other<br>☐ None
If you selected other, please specify what side effects you experienced while taking ondansetron (e.g. Zofran®).	__________________________________
Did you stop taking ondansetron (e.g. Zofran®) because of side effects?	◯ Yes<br>◯ No
Did you ever want to stop taking ondansetron (e.g. Zofran®) because of side effects?	◯ Yes<br>◯ No
How effective do you feel ondansetron (e.g. Zofran®) was in managing your severe nausea and vomiting in pregnancy? (1 being not very effective and 5 being very effective)	◯ 1<br>◯ 2<br>◯ 3<br>◯ 4<br>◯ 5
Would you use ondansetron (e.g. Zofran®) again?	◯ Yes<br>◯ No
Would you recommend ondansetron (e.g. Zofran®) to a friend?	◯ Yes<br>◯ No
If not, why would you not recommend ondansetron (e.g. Zofran®)?	☐ Too expensive<br>☐ Side effects<br>☐ Does not work<br>☐ Other
If you selected other, please specify why you would not recommend ondansetron (e.g. Zofran®).	__________________________________

The following questions relate to your use of metoclopramide (e.g. Maxolon®, Pramin®) during pregnancy for managing nausea and vomiting of pregnancy

Who recommended/prescribed metoclopramide (e.g. Maxolon®, Pramin®) to you?

- ☐ General Practitioner (GP)
- ☐ Obstetrician/Gynaecologist
- ☐ Doctor in Emergency Department or Hospital
- ☐ Specialist Doctor (e.g. obstetric medicine, endocrinology)
- ☐ Midwife
- ☐ Nurse
- ☐ Community Pharmacist
- ☐ Hospital Pharmacist
- ☐ Medicines Information Telephone Service (e.g. MotherSafe)
- ☐ Naturopath
- ☐ Family/friends
- ☐ Internet
- ☐ Social Media
- ☐ Other

If you selected other, please specify who recommended/prescribed metoclopramide (e.g. Maxolon®, Pramin®) to you.

____

When did you start taking metoclopramide (e.g. Maxolon®, Pramin®) during your pregnancy?

____
(Which week of your pregnancy?)

Are you still using metoclopramide (e.g. Maxolon®, Pramin®)?

- ◯ Yes
- ◯ No

If yes, how long have you been taking metoclopramide (e.g. Maxolon®, Pramin®)?

____
(How many weeks?)

If no, how long did you take metoclopramide (e.g. Maxolon®, Pramin®) for?

____
(How many weeks?)

How did you use metoclopramide (e.g. Maxolon®, Pramin®)?

- ☐ Tablet/capsule
- ☐ Injection

(Select all that apply)

What was the highest number of metoclopramide (e.g. Maxolon®, Pramin®) tablets/capsules that you took in a single day?

____

Did you experience any of the following side effects while taking metoclopramide (e.g. Maxolon®, Pramin®)? (tick all that apply)	☐ Headache<br>☐ Muscle/body weakness<br>☐ Numbness/pins-and-needles in hands or feet<br>☐ Stomach cramps<br>☐ Heartburn/indigestion<br>☐ Constipation<br>☐ Diarrhoea<br>☐ Impaired alertness, cognition, memory<br>☐ Sedation (e.g. sleepiness)<br>☐ Worsening nausea/vomiting<br>☐ Dry mouth<br>☐ Blurred vision<br>☐ Other<br>☐ None
If you selected other, please specify what side effects you experienced while taking metoclopramide (e.g. Maxolon®, Pramin®).	__________________________________
Did you stop taking metoclopramide (e.g. Maxolon®, Pramin®) because of side effects?	◯ Yes<br>◯ No
Did you ever want to stop taking metoclopramide (e.g. Maxolon®, Pramin®) because of side effects?	◯ Yes<br>◯ No
How effective do you feel metoclopramide (e.g. Maxolon®, Pramin®) was in managing your severe nausea and vomiting in pregnancy? (1 being not very effective and 5 being very effective)	◯ 1<br>◯ 2<br>◯ 3<br>◯ 4<br>◯ 5
Would you use metoclopramide (e.g. Maxolon®, Pramin®) again?	◯ Yes<br>◯ No
Would you recommend metoclopramide (e.g. Maxolon®, Pramin®) to a friend?	◯ Yes<br>◯ No
If not, why would you not recommend metoclopramide (e.g. Maxolon®, Pramin®)?	☐ Too expensive<br>☐ Side effects<br>☐ Does not work<br>☐ Other
If you selected other, please specify why you would not recommend metoclopramide (e.g. Maxolon®, Pramin®).	__________________________________

The following questions relate to your use of domperidone (e.g. Motilium®) during pregnancy for managing nausea and vomiting of pregnancy

Who recommended/prescribed domperidone (e.g. Motilium®) to you?

- ☐ General Practitioner (GP)
- ☐ Obstetrician/Gynaecologist
- ☐ Doctor in Emergency Department or Hospital
- ☐ Specialist Doctor (e.g. obstetric medicine, endocrinology)
- ☐ Midwife
- ☐ Nurse
- ☐ Community Pharmacist
- ☐ Hospital Pharmacist
- ☐ Medicines Information Telephone Service (e.g. MotherSafe)
- ☐ Naturopath
- ☐ Family/friends
- ☐ Internet
- ☐ Social Media
- ☐ Other

If you selected other, please specify who recommended/prescribed domperidone (e.g. Motilium®) to you.

____

When did you start taking domperidone (e.g. Motilium®) during your pregnancy?

____
(Which week of your pregnancy?)

Are you still using domperidone (e.g. Motilium®)?

- ◯ Yes
- ◯ No

If yes, how long have you been taking domperidone (e.g. Motilium®)?

____
(How many weeks?)

If no, how long did you take domperidone (e.g. Motilium®) for?

____
(How many weeks?)

What was the highest number of domperidone (e.g. Motilium®) tablets/capsules that you took in a single day?

____

Did you experience any of the following side effects while taking domperidone (e.g. Motilium®)? (tick all that apply)

- ☐ Headache
- ☐ Muscle/body weakness
- ☐ Numbness/pins-and-needles in hands or feet
- ☐ Stomach cramps
- ☐ Heartburn/indigestion
- ☐ Constipation
- ☐ Diarrhoea
- ☐ Impaired alertness, cognition, memory
- ☐ Sedation (e.g. sleepiness)
- ☐ Worsening nausea/vomiting
- ☐ Dry mouth
- ☐ Blurred vision
- ☐ Other
- ☐ None

If you selected other, please specify what side effects you experienced while taking domperidone (e.g. Motilium®).	__________________________
Did you stop taking domperidone (e.g. Motilium®) because of side effects?	◯ Yes<br>◯ No
Did you ever want to stop taking domperidone (e.g. Motilium®) because of side effects?	◯ Yes<br>◯ No
How effective do you feel domperidone (e.g. Motilium®) was in managing your severe nausea and vomiting in pregnancy? (1 being not very effective and 5 being very effective)	◯ 1<br>◯ 2<br>◯ 3<br>◯ 4<br>◯ 5
Would you use domperidone (e.g. Motilium®) again?	◯ Yes<br>◯ No
Would you recommend domperidone (e.g. Motilium®) to a friend?	◯ Yes<br>◯ No
If not, why would you not recommend domperidone (e.g. Motilium®)?	☐ Too expensive<br>☐ Side effects<br>☐ Does not work<br>☐ Other
If you selected other, please specify why you would not recommend domperidone (e.g. Motilium®).	__________________________

The following questions relate to your use of mirtazapine (e.g. Avanza®) during pregnancy for managing nausea and vomiting of pregnancy

Who recommended/prescribed mirtazapine (e.g. Avanza®) to you?

- ☐ General Practitioner (GP)
- ☐ Obstetrician/Gynaecologist
- ☐ Doctor in Emergency Department or Hospital
- ☐ Specialist Doctor (e.g. obstetric medicine, endocrinology)
- ☐ Midwife
- ☐ Nurse
- ☐ Community Pharmacist
- ☐ Hospital Pharmacist
- ☐ Medicines Information Telephone Service (e.g. MotherSafe)
- ☐ Naturopath
- ☐ Family/friends
- ☐ Internet
- ☐ Social Media
- ☐ Other

If you selected other, please specify who recommended/prescribed mirtazapine (e.g. Avanza®) to you.

____

When did you start taking mirtazapine (e.g. Avanza®) during your pregnancy?

____
(Which week of your pregnancy?)

Are you still using mirtazapine (e.g. Avanza®)?

- ◯ Yes
- ◯ No

If yes, how long have you been taking mirtazapine (e.g. Avanza®)?

____
(How many weeks?)

If no, how long did you take mirtazapine (e.g. Avanza®) for?

____
(How many weeks?)

What was the highest number of mirtazapine (e.g. Avanza®) tablets/capsules that you took in a single day?

____

What was the dose of each mirtazapine (e.g. Avanza®) tablet/capsule?

- ◯ 15 mg
- ◯ 30 mg
- ◯ 45 mg
- ◯ Don't know

Did you experience any of the following side effects while taking mirtazapine (e.g. Avanza®)? (tick all that apply)	☐ Headache<br>☐ Muscle/body weakness<br>☐ Numbness/pins-and-needles in hands or feet<br>☐ Stomach cramps<br>☐ Heartburn/indigestion<br>☐ Constipation<br>☐ Diarrhoea<br>☐ Impaired alertness, cognition, memory<br>☐ Sedation (e.g. sleepiness)<br>☐ Worsening nausea/vomiting<br>☐ Dry mouth<br>☐ Blurred vision<br>☐ Other<br>☐ None
If you selected other, please specify what side effects you experienced while taking mirtazapine (e.g. Avanza®).	______________________________
Did you stop taking mirtazapine (e.g. Avanza®) because of side effects?	◯ Yes<br>◯ No
Did you ever want to stop taking mirtazapine (e.g. Avanza®) because of side effects?	◯ Yes<br>◯ No
How effective do you feel mirtazapine (e.g. Avanza®) was in managing your severe nausea and vomiting in pregnancy? (1 being not very effective and 5 being very effective)	◯ 1<br>◯ 2<br>◯ 3<br>◯ 4<br>◯ 5
Would you use mirtazapine (e.g. Avanza®) again?	◯ Yes<br>◯ No
Would you recommend mirtazapine (e.g. Avanza®) to a friend?	◯ Yes<br>◯ No
If not, why would you not recommend mirtazapine (e.g. Avanza®)?	☐ Too expensive<br>☐ Side effects<br>☐ Does not work<br>☐ Other
If you selected other, please specify why you not recommend mirtazapine (e.g. Avanza®).	______________________________

The following questions relate to your use of steroids (e.g. prednisolone [Panafcortelone®, Solone]) during pregnancy for managing nausea and vomiting of pregnancy

Who recommended/prescribed steroids (e.g. prednisolone [Panafcortelone®, Solone]) to you?	☐ General Practitioner (GP)<br>☐ Obstetrician/Gynaecologist<br>☐ Doctor in Emergency Department or Hospital<br>☐ Specialist Doctor (e.g. obstetric medicine, endocrinology)<br>☐ Midwife<br>☐ Nurse<br>☐ Community Pharmacist<br>☐ Hospital Pharmacist<br>☐ Medicines Information Telephone Service (e.g. MotherSafe)<br>☐ Naturopath<br>☐ Family/friends<br>☐ Internet<br>☐ Social Media<br>☐ Other
If you selected other, please specify who recommended/prescribed steroids (e.g. prednisolone [Panafcortelone®, Solone]) to you.	______________________________
When did you start taking steroids (e.g. prednisolone [Panafcortelone®, Solone]) during your pregnancy?	______________________________<br>(Which week of your pregnancy?)
Are you still using steroids (e.g. prednisolone [Panafcortelone®, Solone])?	◯ Yes<br>◯ No
If yes, how long have you been taking steroids (e.g. prednisolone [Panafcortelone®, Solone])?	______________________________<br>(How many weeks?)
If no, how long did you take steroids (e.g. prednisolone [Panafcortelone®, Solone]) for?	______________________________<br>(How many weeks?)
How did you use steroids (e.g. prednisolone [Panafcortelone®, Solone])?	☐ Tablet/capsule<br>☐ Injection<br>☐ Liquid<br>(Select all that apply)
What was the highest amount of steroid (e.g. prednisolone [Panafcortelone®, Solone]) tablets/capsules that you took in one day?	______________________________
What was the dose of each steroid (e.g. prednisolone [Panafcortelone®, Solone]) capsule/tablet?	◯ 25 mg<br>◯ Other<br>◯ Don't Know
If you selected other, please specify the dose of each steroid (e.g. prednisolone [Panafcortelone®, Solone]) capsule/tablet.	______________________________

Did you experience any of the following side effects while taking steroids (e.g. prednisolone [Panafcortelone®, Solone])? (tick all that apply)	☐ Headache<br>☐ Muscle/body weakness<br>☐ Numbness/pins-and-needles in hands or feet<br>☐ Stomach cramps<br>☐ Heartburn/indigestion<br>☐ Constipation<br>☐ Diarrhoea<br>☐ Impaired alertness, cognition, memory<br>☐ Sedation (e.g. sleepiness)<br>☐ Worsening nausea/vomiting<br>☐ Dry mouth<br>☐ Blurred vision<br>☐ Other<br>☐ None
If you selected other, please specify what side effects you experienced while taking steroids (e.g. prednisolone [Panafcortelone®, Solone]).	__________________________
Did you stop taking steroids (e.g. prednisolone [Panafcortelone®, Solone]) because of side effects?	◯ Yes<br>◯ No
Did you ever want to stop taking steroids (e.g. prednisolone [Panafcortelone®, Solone]) because of side effects?	◯ Yes<br>◯ No
How effective do you feel steroids (e.g. prednisolone [Panafcortelone®, Solone]) was in managing your severe nausea and vomiting in pregnancy? (1 being not very effective and 5 being very effective)	◯ 1<br>◯ 2<br>◯ 3<br>◯ 4<br>◯ 5
Would you use steroids (e.g. prednisolone [Panafcortelone®, Solone]) again?	◯ Yes<br>◯ No
Would you recommend steroids (e.g. prednisolone [Panafcortelone®, Solone]) to a friend?	◯ Yes<br>◯ No
If not, why would you not recommend steroids (e.g. prednisolone [Panafcortelone®, Solone])?	☐ Too expensive<br>☐ Side effects<br>☐ Does not work<br>☐ Other
If you selected other, please specify why you would not recommend steroids (e.g. prednisolone [Panafcortelone®, Solone]).	__________________________

The following questions relate to your use of antacids (e.g. Mylanta®, Gaviscon®) during pregnancy for managing nausea and vomiting of pregnancy

Who recommended/prescribed antacids (e.g. Mylanta®, Gaviscon®) to you?

- ☐ General Practitioner (GP)
- ☐ Obstetrician/Gynaecologist
- ☐ Doctor in Emergency Department or Hospital
- ☐ Specialist Doctor (e.g. obstetric medicine, endocrinology)
- ☐ Midwife
- ☐ Nurse
- ☐ Community Pharmacist
- ☐ Hospital Pharmacist
- ☐ Medicines Information Telephone Service (e.g. MotherSafe)
- ☐ Naturopath
- ☐ Family/friends
- ☐ Internet
- ☐ Social Media
- ☐ Other

If you selected other, please specify who recommended/prescribed antacids (e.g. Mylanta®, Gaviscon®) to you.

____

When did you start taking antacids (e.g. Mylanta®, Gaviscon®) during your pregnancy?

____
(Which week of your pregnancy?)

Are you still using antacids (e.g. Mylanta®, Gaviscon®)?

- ◯ Yes
- ◯ No

If yes, how long have you been taking antacids (e.g. Mylanta®, Gaviscon®)?

____
(How many weeks?)

If no, how long did you take antacids (e.g. Mylanta®, Gaviscon®) for?

____
(How many weeks?)

Did you experience any of the following side effects while taking antacids (e.g. Mylanta®, Gaviscon®)? (tick all that apply)

- ☐ Headache
- ☐ Muscle/body weakness
- ☐ Numbness/pins-and-needles in hands or feet
- ☐ Stomach cramps
- ☐ Heartburn/indigestion
- ☐ Constipation
- ☐ Diarrhoea
- ☐ Impaired alertness, cognition, memory
- ☐ Sedation (e.g. sleepiness)
- ☐ Worsening nausea/vomiting
- ☐ Dry mouth
- ☐ Blurred vision
- ☐ Other
- ☐ None

If you selected other, please specify what side effects you experienced while taking antacids (e.g. Mylanta®, Gaviscon®).

____

Did you stop taking antacids (e.g. Mylanta®, Gaviscon®) because of side effects?

- ◯ Yes
- ◯ No

Did you ever want to stop taking antacids (e.g. Mylanta®, Gaviscon®) because of side effects?	◯ Yes<br>◯ No
How effective do you feel antacids (e.g. Mylanta®, Gaviscon®) was in managing your severe nausea and vomiting in pregnancy? (1 being not very effective and 5 being very effective)	◯ 1<br>◯ 2<br>◯ 3<br>◯ 4<br>◯ 5
Would you use antacids (e.g. Mylanta®, Gaviscon®) again?	◯ Yes<br>◯ No
Would you recommend antacids (e.g. Mylanta®, Gaviscon®) to a friend?	◯ Yes<br>◯ No
If not, why would you not recommend antacids (e.g. Mylanta®, Gaviscon®)?	☐ Too expensive<br>☐ Side effects<br>☐ Does not work<br>☐ Other
If you selected other, please specify why you would not recommend antacids (e.g. Mylanta®, Gaviscon®).	__________________________

The following questions relate to your use of H2 antagonist (e.g. ranitidine [Zantac™]) during pregnancy for managing nausea and vomiting of pregnancy

Who recommended/prescribed H2 antagonist (e.g. ranitidine [Zantac™]) to you?

- ☐ General Practitioner (GP)
- ☐ Obstetrician/Gynaecologist
- ☐ Doctor in Emergency Department or Hospital
- ☐ Specialist Doctor (e.g. obstetric medicine, endocrinology)
- ☐ Midwife
- ☐ Nurse
- ☐ Community Pharmacist
- ☐ Hospital Pharmacist
- ☐ Medicines Information Telephone Service (e.g. MotherSafe)
- ☐ Naturopath
- ☐ Family/friends
- ☐ Internet
- ☐ Social Media
- ☐ Other

If you selected other, please specify who recommended/prescribed H2 antagonist (e.g. ranitidine [Zantac™]) to you.

____

When did you start taking H2 antagonist (e.g. ranitidine [Zantac™]) during your pregnancy?

____
(Which week of your pregnancy?)

Are you still using H2 antagonist (e.g. ranitidine [Zantac™])?

- ◯ Yes
- ◯ No

If yes, how long have you been taking H2 antagonist (e.g. ranitidine [Zantac™])?

____
(How many weeks?)

If no, how long did you take H2 antagonist (e.g. ranitidine [Zantac™]) for?

____
(How many weeks?)

Did you experience any of the following side effects while taking H2 antagonist (e.g. ranitidine [Zantac™])? (tick all that apply)

- ☐ Headache
- ☐ Muscle/body weakness
- ☐ Numbness/pins-and-needles in hands or feet
- ☐ Stomach cramps
- ☐ Heartburn/indigestion
- ☐ Constipation
- ☐ Diarrhoea
- ☐ Impaired alertness, cognition, memory
- ☐ Sedation (e.g. sleepiness)
- ☐ Worsening nausea/vomiting
- ☐ Dry mouth
- ☐ Blurred vision
- ☐ Other
- ☐ None

If you selected other, please specify what side effects you experienced while taking H2 antagonist (e.g. ranitidine [Zantac™]).

____

Did you stop taking H2 antagonist (e.g. ranitidine [Zantac™]) because of side effects?

- ◯ Yes
- ◯ No

Did you ever want to stop taking H2 antagonist (e.g. ranitidine [Zantac™]) because of side effects?	◯ Yes<br>◯ No
How effective do you feel H2 antagonist (e.g. ranitidine [Zantac™]) was in managing your severe nausea and vomiting in pregnancy? (1 being not very effective and 5 being very effective)	◯ 1<br>◯ 2<br>◯ 3<br>◯ 4<br>◯ 5
Would you use H2 antagonist (e.g. ranitidine [Zantac™]) again?	◯ Yes<br>◯ No
Would you recommend H2 antagonist (e.g. ranitidine [Zantac™]) to a friend?	◯ Yes<br>◯ No
If not, why would you not recommend H2 antagonist (e.g. ranitidine [Zantac™])?	☐ Too expensive<br>☐ Side effects<br>☐ Does not work<br>☐ Other
If you selected other, please specify why you would not recommend H2 antagonist (e.g. ranitidine [Zantac™]).	__________________________

The following questions relate to your use of proton pump inhibitor (e.g. omeprazole [Losec®, Acimax®]) during pregnancy for managing nausea and vomiting of pregnancy

Who recommended/prescribed proton pump inhibitor (e.g. omeprazole [Losec®, Acimax®]) to you?

- ☐ General Practitioner (GP)
- ☐ Obstetrician/Gynaecologist
- ☐ Doctor in Emergency Department or Hospital
- ☐ Specialist Doctor (e.g. obstetric medicine, endocrinology)
- ☐ Midwife
- ☐ Nurse
- ☐ Community Pharmacist
- ☐ Hospital Pharmacist
- ☐ Medicines Information Telephone Service (e.g. MotherSafe)
- ☐ Naturopath
- ☐ Family/friends
- ☐ Internet
- ☐ Social Media
- ☐ Other

If you selected other, please specify who recommended/prescribed proton pump inhibitor (e.g. omeprazole [Losec®, Acimax®]) to you.

____

When did you start taking proton pump inhibitor (e.g. omeprazole [Losec®, Acimax®]) during your pregnancy?

____
(Which week of your pregnancy?)

Are you still using proton pump inhibitor (e.g. omeprazole [Losec®, Acimax®])?

- ○ Yes
- ○ No

If yes, how long have you been taking proton pump inhibitor (e.g. omeprazole [Losec®, Acimax®])?

____
(How many weeks?)

If no, how long did you take proton pump inhibitor (e.g. omeprazole [Losec®, Acimax®]) for?

____
(How many weeks?)

Did you experience any of the following side effects while taking proton pump inhibitor (e.g. omeprazole [Losec®, Acimax®])? (tick all that apply)

- ☐ Headache
- ☐ Muscle/body weakness
- ☐ Numbness/pins-and-needles in hands or feet
- ☐ Stomach cramps
- ☐ Heartburn/indigestion
- ☐ Constipation
- ☐ Diarrhoea
- ☐ Impaired alertness, cognition, memory
- ☐ Sedation (e.g. sleepiness)
- ☐ Worsening nausea/vomiting
- ☐ Dry mouth
- ☐ Blurred vision
- ☐ Other
- ☐ None

If you selected other, please specify what side effects you experienced while taking proton pump inhibitor (e.g. omeprazole [Losec®, Acimax®]).

____

Did you stop taking proton pump inhibitor (e.g. omeprazole [Losec®, Acimax®]) because of side effects?	○ Yes<br>○ No
Did you ever want to stop taking proton pump inhibitor (e.g. omeprazole [Losec®, Acimax®]) because of side effects?	○ Yes<br>○ No
How effective do you feel proton pump inhibitor (e.g. omeprazole [Losec®, Acimax®]) was in managing your severe nausea and vomiting in pregnancy? (1 being not very effective and 5 being very effective)	○ 1<br>○ 2<br>○ 3<br>○ 4<br>○ 5
Would you use proton pump inhibitor (e.g. omeprazole [Losec®, Acimax®]) again?	○ Yes<br>○ No
Would you recommend proton pump inhibitor (e.g. omeprazole [Losec®, Acimax®]) to a friend?	○ Yes<br>○ No
If not, why would you not recommend proton pump inhibitor (e.g. omeprazole [Losec®, Acimax®])?	☐ Too expensive<br>☐ Side effects<br>☐ Does not work<br>☐ Other
If you selected other, please specify why you would not recommend proton pump inhibitor (e.g. omeprazole [Losec®, Acimax®]).	______________________

The following questions relate to your use of cannabis/marijuana during pregnancy for managing nausea and vomiting of pregnancy

Who recommended/prescribed cannabis/marijuana to you?

- ☐ General Practitioner (GP)
- ☐ Obstetrician/Gynaecologist
- ☐ Doctor in Emergency Department or Hospital
- ☐ Specialist Doctor (e.g. obstetric medicine, endocrinology)
- ☐ Midwife
- ☐ Nurse
- ☐ Community Pharmacist
- ☐ Hospital Pharmacist
- ☐ Medicines Information Telephone Service (e.g. MotherSafe)
- ☐ Naturopath
- ☐ Family/friends
- ☐ Internet
- ☐ Social Media
- ☐ Other

If you selected other, please specify who recommended/prescribed cannabis/marijuana to you.

____

When did you start taking cannabis/marijuana during your pregnancy?

____
(Which week of your pregnancy?)

Are you still using cannabis/marijuana?

- ◯ Yes
- ◯ No

If yes, how long have you been taking cannabis/marijuana?

____
(How many weeks?)

If no, how long did you take cannabis/marijuana for?

____
(How many weeks?)

How did you use cannabis?

____
(e.g. smoked, vaped, eaten)

What was the highest number of times you used cannabis/marijuana in one day?

____

Did you experience any of the following side effects while taking cannabis/marijuana? (tick all that apply)

- ☐ Headache
- ☐ Muscle/body weakness
- ☐ Numbness/pins-and-needles in hands or feet
- ☐ Stomach cramps
- ☐ Heartburn/indigestion
- ☐ Constipation
- ☐ Diarrhoea
- ☐ Impaired alertness, cognition, memory
- ☐ Sedation (e.g. sleepiness)
- ☐ Worsening nausea/vomiting
- ☐ Dry mouth
- ☐ Blurred vision
- ☐ Other
- ☐ None

If you selected other, please specify what side effects you experienced while taking cannabis/marijuana.	__________________________________
Did you stop taking cannabis/marijuana because of side effects?	◯ Yes<br>◯ No
Did you ever want to stop taking cannabis/marijuana because of side effects?	◯ Yes<br>◯ No
How effective do you feel cannabis/marijuana was in managing your severe nausea and vomiting in pregnancy? (1 being not very effective and 5 being very effective)	◯ 1<br>◯ 2<br>◯ 3<br>◯ 4<br>◯ 5
Would you use cannabis/marijuana again?	◯ Yes<br>◯ No
Would you recommend cannabis/marijuana to a friend?	◯ Yes<br>◯ No
If not, why would you not recommend cannabis/marijuana?	☐ Too expensive<br>☐ Side effects<br>☐ Does not work<br>☐ Other
If you selected other, please specify why you would not recommend cannabis/marijuana.	__________________________________

The following questions relate to your use of acupressure (e.g. SeaBand®) during pregnancy for managing nausea and vomiting of pregnancy

Who recommended acupressure (e.g. SeaBand®) to you?
- ☐ General Practitioner (GP)
- ☐ Obstetrician/Gynaecologist
- ☐ Doctor in Emergency Department or Hospital
- ☐ Specialist Doctor (e.g. obstetric medicine, endocrinology)
- ☐ Midwife
- ☐ Nurse
- ☐ Community Pharmacist
- ☐ Hospital Pharmacist
- ☐ Medicines Information Telephone Service (e.g. MotherSafe)
- ☐ Naturopath
- ☐ Family/friends
- ☐ Internet
- ☐ Social Media
- ☐ Other

If you selected other, please specify who recommended acupressure (e.g. SeaBand®) to you.

____

When did you start using acupressure (e.g. SeaBand®) during your pregnancy?

____
(Which week of your pregnancy?)

Are you still using acupressure (e.g. SeaBand®)?
- ◯ Yes
- ◯ No

If yes, how long have you been using acupressure (e.g. SeaBand®)?

____
(How many weeks?)

If no, how long did you use acupressure (e.g. SeaBand®) for?

____
(How many weeks?)

Did you experience any of the following side effects while using acupressure (e.g. SeaBand®)? (tick all that apply)
- ☐ Headache
- ☐ Muscle/body weakness
- ☐ Numbness/pins-and-needles in hands or feet
- ☐ Stomach cramps
- ☐ Heartburn/indigestion
- ☐ Constipation
- ☐ Diarrhoea
- ☐ Impaired alertness, cognition, memory
- ☐ Sedation (e.g. sleepiness)
- ☐ Worsening nausea/vomiting
- ☐ Dry mouth
- ☐ Blurred vision
- ☐ Other
- ☐ None

If you selected other, please specify what side effects you experienced while using acupressure (e.g. SeaBand®).

____

Did you stop using acupressure (e.g. SeaBand®) because of side effects?
- ◯ Yes
- ◯ No

Did you ever want to stop using acupressure (e.g. SeaBand®) because of side effects?	◯ Yes<br>◯ No
How effective do you feel acupressure (e.g. SeaBand®) was in managing your severe nausea and vomiting in pregnancy? (1 being not very effective and 5 being very effective)	◯ 1<br>◯ 2<br>◯ 3<br>◯ 4<br>◯ 5
Would you use acupressure (e.g. SeaBand®) again?	◯ Yes<br>◯ No
Would you recommend acupressure (e.g. SeaBand®) to a friend?	◯ Yes<br>◯ No
If not, why would you not recommend acupressure (e.g. SeaBand®)?	☐ Too expensive<br>☐ Side effects<br>☐ Does not work<br>☐ Other
If you selected other, please specify why you would not recommend acupressure (e.g. SeaBand®).	__________________________________

The following questions relate to your use of acupuncture during pregnancy for managing nausea and vomiting of pregnancy

Who recommended acupuncture to you?

- ☐ General Practitioner (GP)
- ☐ Obstetrician/Gynaecologist
- ☐ Doctor in Emergency Department or Hospital
- ☐ Specialist Doctor (e.g. obstetric medicine, endocrinology)
- ☐ Midwife
- ☐ Nurse
- ☐ Community Pharmacist
- ☐ Hospital Pharmacist
- ☐ Medicines Information Telephone Service (e.g. MotherSafe)
- ☐ Naturopath
- ☐ Family/friends
- ☐ Internet
- ☐ Social Media
- ☐ Other

If you selected other, please specify who recommended acupuncture to you.

____

When did you start receiving acupuncture during your pregnancy?

____
(Which week of your pregnancy?)

Are you still receiving acupuncture?

- ◯ Yes
- ◯ No

If yes, how long have you been receiving acupuncture?

____
(How many weeks?)

If no, how long did you receive acupuncture for?

____
(How many weeks?)

Did you experience any of the following side effects while receiving acupuncture? (tick all that apply)

- ☐ Headache
- ☐ Muscle/body weakness
- ☐ Numbness/pins-and-needles in hands or feet
- ☐ Stomach cramps
- ☐ Heartburn/indigestion
- ☐ Constipation
- ☐ Diarrhoea
- ☐ Impaired alertness, cognition, memory
- ☐ Sedation (e.g. sleepiness)
- ☐ Worsening nausea/vomiting
- ☐ Dry mouth
- ☐ Blurred vision
- ☐ Other
- ☐ None

If you selected other, please specify what side effects you experienced while using acupuncture.

____

Did you stop receiving acupuncture because of side effects?

- ◯ Yes
- ◯ No

Did you ever want to stop receiving acupuncture because of side effects?	◯ Yes<br>◯ No
How effective do you feel acupuncture was in managing your severe nausea and vomiting in pregnancy? (1 being not very effective and 5 being very effective)	◯ 1<br>◯ 2<br>◯ 3<br>◯ 4<br>◯ 5
Would you use acupuncture again?	◯ Yes<br>◯ No
Would you recommend acupuncture to a friend?	◯ Yes<br>◯ No
If not, why would you not recommend acupuncture?	☐ Too expensive<br>☐ Side effects<br>☐ Does not work<br>☐ Other
If you selected other, please specify why you would not recommend acupuncture.	__________________________________

The following questions relate to your use of homeopathy during pregnancy for managing nausea and vomiting of pregnancy

Who recommended homeopathy to you?

- ☐ General Practitioner (GP)
- ☐ Obstetrician/Gynaecologist
- ☐ Doctor in Emergency Department or Hospital
- ☐ Specialist Doctor (e.g. obstetric medicine, endocrinology)
- ☐ Midwife
- ☐ Nurse
- ☐ Community Pharmacist
- ☐ Hospital Pharmacist
- ☐ Medicines Information Telephone Service (e.g. MotherSafe)
- ☐ Naturopath
- ☐ Family/friends
- ☐ Internet
- ☐ Social Media
- ☐ Other

If you selected other, please specify who recommended homeopathy to you.

____

When did you start using homeopathy during your pregnancy?

____
(Which week of your pregnancy?)

Are you still using homeopathy?

- ◯ Yes
- ◯ No

If yes, how long have you been using homeopathy?

____
(How many weeks?)

If no, how long did you use homeopathy for?

____
(How many weeks?)

Did you experience any of the following side effects while using homeopathy? (tick all that apply)

- ☐ Headache
- ☐ Muscle/body weakness
- ☐ Numbness/pins-and-needles in hands or feet
- ☐ Stomach cramps
- ☐ Heartburn/indigestion
- ☐ Constipation
- ☐ Diarrhoea
- ☐ Impaired alertness, cognition, memory
- ☐ Sedation (e.g. sleepiness)
- ☐ Worsening nausea/vomiting
- ☐ Dry mouth
- ☐ Blurred vision
- ☐ Other
- ☐ None

If you selected other, please specify what side effects you experienced while using homeopathy.

____

Did you stop using homeopathy because of side effects?

- ◯ Yes
- ◯ No

Did you ever want to stop using homeopathy because of side effects?	◯ Yes<br>◯ No
How effective do you feel homeopathy was in managing your severe nausea and vomiting in pregnancy? (1 being not very effective and 5 being very effective)	◯ 1<br>◯ 2<br>◯ 3<br>◯ 4<br>◯ 5
Would you use homeopathy again?	◯ Yes<br>◯ No
Would you recommend homeopathy to a friend?	◯ Yes<br>◯ No
If not, why would you not recommend homeopathy?	☐ Too expensive<br>☐ Side effects<br>☐ Does not work<br>☐ Other
If you selected other, please specify why you would not recommend homeopathy.	__________________________________

The following questions relate to your use of aromatherapy during pregnancy for managing nausea and vomiting of pregnancy

Who recommended aromatherapy to you?

- ☐ General Practitioner (GP)
- ☐ Obstetrician/Gynaecologist
- ☐ Doctor in Emergency Department or Hospital
- ☐ Specialist Doctor (e.g. obstetric medicine, endocrinology)
- ☐ Midwife
- ☐ Nurse
- ☐ Community Pharmacist
- ☐ Hospital Pharmacist
- ☐ Medicines Information Telephone Service (e.g. MotherSafe)
- ☐ Naturopath
- ☐ Family/friends
- ☐ Internet
- ☐ Social Media
- ☐ Other

If you selected other, please specify who recommended aromatherapy to you.

____

When did you start using aromatherapy during your pregnancy?

____
(Which week of your pregnancy?)

Are you still using aromatherapy?

- ◯ Yes
- ◯ No

If yes, how long have you been using aromatherapy?

____
(How many weeks?)

If no, how long did you use aromatherapy for?

____
(How many weeks?)

Did you experience any of the following side effects while using aromatherapy? (tick all that apply)

- ☐ Headache
- ☐ Muscle/body weakness
- ☐ Numbness/pins-and-needles in hands or feet
- ☐ Stomach cramps
- ☐ Heartburn/indigestion
- ☐ Constipation
- ☐ Diarrhoea
- ☐ Impaired alertness, cognition, memory
- ☐ Sedation (e.g. sleepiness)
- ☐ Worsening nausea/vomiting
- ☐ Dry mouth
- ☐ Blurred vision
- ☐ Other
- ☐ None

If you selected other, please specify what side effects you experienced while using aromatherapy.

____

Did you stop using aromatherapy because of side effects?

- ◯ Yes
- ◯ No

Did you ever want to stop using aromatherapy because of side effects?	◯ Yes<br>◯ No
How effective do you feel aromatherapy was in managing your severe nausea and vomiting in pregnancy? (1 being not very effective and 5 being very effective)	◯ 1<br>◯ 2<br>◯ 3<br>◯ 4<br>◯ 5
Would you use aromatherapy again?	◯ Yes<br>◯ No
Would you recommend aromatherapy to a friend?	◯ Yes<br>◯ No
If not, why would you not recommend aromatherapy?	☐ Too expensive<br>☐ Side effects<br>☐ Does not work<br>☐ Other
If you selected other, please specify why you would not recommend aromatherapy.	__________________________________

The following questions relate to your use of multivitamins during pregnancy for managing nausea and vomiting of pregnancy

Who recommended multivitamins to you?

- ☐ General Practitioner (GP)
- ☐ Obstetrician/Gynaecologist
- ☐ Doctor in Emergency Department or Hospital
- ☐ Specialist Doctor (e.g. obstetric medicine, endocrinology)
- ☐ Midwife
- ☐ Nurse
- ☐ Community Pharmacist
- ☐ Hospital Pharmacist
- ☐ Medicines Information Telephone Service (e.g. MotherSafe)
- ☐ Naturopath
- ☐ Family/friends
- ☐ Internet
- ☐ Social Media
- ☐ Other

If you selected other, please specify who recommended multivitamins to you.

____

When did you start taking multivitamins during your pregnancy?

____
(Which week of your pregnancy?)

Are you still using multivitamins?

- ◯ Yes
- ◯ No

If yes, how long have you been taking multivitamins?

____
(How many weeks?)

If no, how long did you take multivitamins for?

____
(How many weeks?)

Did you experience any of the following side effects while taking multivitamins? (tick all that apply)

- ☐ Headache
- ☐ Muscle/body weakness
- ☐ Numbness/pins-and-needles in hands or feet
- ☐ Stomach cramps
- ☐ Heartburn/indigestion
- ☐ Constipation
- ☐ Diarrhoea
- ☐ Impaired alertness, cognition, memory
- ☐ Sedation (e.g. sleepiness)
- ☐ Worsening nausea/vomiting
- ☐ Dry mouth
- ☐ Blurred vision
- ☐ Other
- ☐ None

If you selected other, please specify what side effects you experienced while taking multivitamins.

____

Did you stop taking multivitamins because of side effects?

- ◯ Yes
- ◯ No

Did you ever want to stop taking multivitamins because of side effects?	◯ Yes<br>◯ No
How effective do you feel multivitamins was in managing your severe nausea and vomiting in pregnancy? (1 being not very effective and 5 being very effective)	◯ 1<br>◯ 2<br>◯ 3<br>◯ 4<br>◯ 5
Would you use multivitamins again?	◯ Yes<br>◯ No
Would you recommend multivitamins to a friend?	◯ Yes<br>◯ No
If not, why would you not recommend multivitamins?	☐ Too expensive<br>☐ Side effects<br>☐ Does not work<br>☐ Other
If you selected other, please specify why you would not recommend multivitamins.	__________________________

The following questions relate to your use of herbal therapies during pregnancy for managing nausea and vomiting of pregnancy

Please describe what type of herbal therapies you took during pregnancy
____

Who recommended herbal remedies to you?
- ☐ General Practitioner (GP)
- ☐ Obstetrician/Gynaecologist
- ☐ Doctor in Emergency Department or Hospital
- ☐ Specialist Doctor (e.g. obstetric medicine, endocrinology)
- ☐ Midwife
- ☐ Nurse
- ☐ Community Pharmacist
- ☐ Hospital Pharmacist
- ☐ Medicines Information Telephone Service (e.g. MotherSafe)
- ☐ Naturopath
- ☐ Family/friends
- ☐ Internet
- ☐ Social Media
- ☐ Other

If you selected other, please specify who recommended herbal remedies to you.
____

When did you start taking herbal remedies during your pregnancy?
____
(Which week of your pregnancy?)

Are you still using herbal remedies?
- ◯ Yes
- ◯ No

If yes, how long have you been taking herbal remedies?
____
(How many weeks?)

If no, how long did you take herbal remedies?
____
(How many weeks?)

Did you experience any of the following side effects while taking herbal remedies? (tick all that apply)
- ☐ Headache
- ☐ Muscle/body weakness
- ☐ Numbness/pins-and-needles in hands or feet
- ☐ Stomach cramps
- ☐ Heartburn/indigestion
- ☐ Constipation
- ☐ Diarrhoea
- ☐ Impaired alertness, cognition, memory
- ☐ Sedation (e.g. sleepiness)
- ☐ Worsening nausea/vomiting
- ☐ Dry mouth
- ☐ Blurred vision
- ☐ Other
- ☐ None

If you selected other, please specify what side effects you experienced while taking herbal remedies.
____

Did you stop taking herbal remedies because of side effects?	◯ Yes<br>◯ No
Did you ever want to stop taking herbal remedies because of side effects?	◯ Yes<br>◯ No
How effective do you feel herbal remedies was in managing your severe nausea and vomiting in pregnancy? (1 being not very effective and 5 being very effective)	◯ 1<br>◯ 2<br>◯ 3<br>◯ 4<br>◯ 5
Would you use herbal remedies again?	◯ Yes<br>◯ No
Would you recommend herbal remedies to a friend?	◯ Yes<br>◯ No
If not, why would you not recommend herbal remedies?	☐ Too expensive<br>☐ Side effects<br>☐ Does not work<br>☐ Other
If you selected other, please specify why you would not recommend herbal remedies.	__________________________

Section Five

Were you ever denied a medication by a health care professional because you were pregnant?

◯ Yes
◯ No

If yes, please tell us about this experience

____

Were you ever prescribed or recommended a medication that you refused to take because you did not think it was safe to use during pregnancy?

◯ Yes
◯ No

If yes, please tell us about this experience.

____

Have you heard of the SOMANZ (Society of Obstetric Medicine of Australia and New Zealand) Guideline for the Management of Nausea and Vomiting in Pregnancy and Hyperemesis Gravidarum?

◯ Yes
◯ No

How did you hear about the SOMANZ Guideline for the Management of Nausea and Vomiting in Pregnancy (NVP) and Hyperemesis Gravidarum (HG)? (tick all that apply)

☐ I haven't heard of them
☐ From the website of the charity Hyperemesis Australia
☐ From the support group of the charity Hyperemesis Australia
☐ From an online support group (other than Hyperemesis Australia)
☐ From a health practitioner (e.g. GP, midwife, hospital doctor, community pharmacist)
☐ From an online article
☐ Can't remember
☐ Other

If you selected other, please specify how did you heard about the SOMANZ Guideline for the Management of Nausea and Vomiting in Pregnancy (NVP) and Hyperemesis Gravidarum (HG).

____

How useful did you find the SOMANZ Guidelines to be?

☐ It helped me to understand NVP and HG more
☐ It helped me educate my family and friends about NVP and HG
☐ It helped me educate my GP about NVP and HG
☐ My GP was reluctant to prescribe medication but did so after I showed them the guidelines
☐ It helped reassure me that the medications were safe to take
☐ Other (please specify)

If you selected other, please specify how useful you found the SOMANZ Guidelines to be?

____

This is the final question.

Do you have anything else that you would like to tell us about your thoughts or experiences of using medications or other substances for managing severe nausea and/or vomiting during pregnancy? Feel free to write as much as you like. ____

Thank you for completing this survey.

Your responses will help us to better inform women about different approaches towards managing severe nausea and vomiting in pregnancy.

We welcome you to share this survey with any family, friends or colleagues who you think might be willing to share their experiences of considering medications and other substances for managing severe nausea and vomiting during pregnancy. Given the nature of the questions asked, if you do share the survey we ask that you do so in a sensitive manner that does not cause others embarrassment or distress.

If you would like to share this survey, please email this survey link: https://is.gd/hyperemesis
